# Er,Cr:YSGG Laser Performance Improves Biological Response on Titanium Surfaces

**DOI:** 10.3390/ma13030756

**Published:** 2020-02-07

**Authors:** Wan-Ling Yao, Jerry Chin Yi Lin, Eisner Salamanca, Yu-Hwa Pan, Pei-Yo Tsai, Sy-Jye Leu, Kai-Chiang Yang, Haw-Ming Huang, Huei-Yu Huang, Wei-Jen Chang

**Affiliations:** 1 School of Dentistry, College of Oral Medicine, Taipei Medical University, Taipei 110, Taiwan; yaoyao061637@gmail.com (W.-L.Y.); drjerrylin@gmail.com (J.C.Y.L.); shalom.dc@msa.hinet.net (Y.-H.P.); hhm@tmu.edu.tw (H.-M.H.); 2 Department of General Dentistry, Chang Gung Memorial Hospital, Taipei 106, Taiwan; 3 Graduate Institute of Dental & Craniofacial Science, Chang Gung University, Taoyuan 333, Taiwan; 4 School of Dentistry, College of Medicine, China Medical University, Taichung 404, Taiwan; 5 Department of Microbiology and Immunology, School of Medicine, College of Medicine, Taipei Medical University, Taipei 110, Taiwan; cmbsycl@tmu.edu.tw; 6 School of Dental Technology, College of Oral Medicine, Taipei Medical University, Taipei 110, Taiwan; pumpkin@tmu.edu.tw; 7 Dental Department, Taipei Medical University, Shuang-Ho hospital, Taipei 235, Taiwan

**Keywords:** laser treatment, Er,Cr:YSGG laser, Ti discs, dental implant

## Abstract

*Porphyromonas gingivalis* infection is one of the causes of implant failures, which can lead to peri-implantitis. Implant surface roughness is reportedly related strongly to *P*. *gingivalis* adhesion, which can lead to peri-implantitis and, later, cell adhesion. Our aim was to evaluate the effects of Er,Cr:YSGG laser on titanium (Ti) disc surfaces and its interaction with bacterial adhesion and fibroblast viability. Ti discs underwent two treatments: autoclaving (control) and erbium, chromium-doped yttrium scandium gallium garnet (Er,Cr:YSGG) laser treatment (test). Ti disc surfaces were examined with scanning electronic microscope (SEM), Energy-dispersive spectrometry (EDX), X-ray photoelectron spectroscopy (XPS). The surface roughness same as wettability were also investigated. Fibroblast viability was assessed with the water-soluble tetrazolium 1 (WST-1) test, and osteoblast differentiation was assessed with the alkaline phosphatase (ALP) assay. Bacterial structure and colony formation were detected with scanning electron microscopy and Gram stain. In comparison to control discs, the test discs showed smoother surfaces, with 0.25-µm decrease in surface roughness (*p* < 0.05); lower *P*. *gingivalis* adhesion (*p* < 0.01); less *P*. *gingivalis* colonization (*p* < 0.05); and increased fibroblast viability and osteoblast differentiation (*p* < 0.05). Er,Cr:YSGG laser treatment improved disc surfaces by making them slightly smoother, which reduced *P. gingivalis* adhesion and increased fibroblast viability and osteoblast differentiation. Er,Cr:YSGG laser treatment can be considered a good option for managing peri-implantitis. Further investigations of laser-assisted therapy are necessary for better guidelines in the treatment of peri-implantitis.

## 1. Introduction

Dental implants represent a breakthrough in dentistry [1,2,3]. Annually, 12 to 18 million dental implants are estimated to be sold on a global scale [4,5]. The increasing use of dental implants and the longer follow-up periods have shown an increased incidence of peri-implant mucositis and peri-implantitis, diseases that have become targets of prevention and treatment in daily dental practice. Titanium (Ti) has been considered the suitable material for dental implants because of its osseointegration capabilities and relatively innocuous relationship with the surrounding soft tissues [6,7]. Sometimes accumulation of dental plaque after dental implantation causes complications, such as infections, peri-implant mucositis, and peri-implantitis [8,9,10]. According to the 2017 World Workshop of the American Academy of Periodontology and European Federation of Periodontology, peri-implant mucositis is an inflammatory lesion of the peri-implant mucosa in the absence of continuing marginal bone loss. Peri-implant mucositis is caused primarily by a disruption of host–microbe homeostasis at the implant–mucosa interface and is a reversible condition [11]. This lesion can transform into peri-implantitis, which in the 2017 World Workshop was defined as a pathological condition occurring in tissues around dental implants, characterized by inflammation in the peri-implant connective tissue and by progressive loss of supporting bone [12].

A major cause of peri-implantitis is microorganism accumulation in form of biofilm, which, depending on its composition and other host factors, can induce the inflammation and damage of the oral tissues, as described by the 2017 World Workshop [13,14,15]. An increasing number of anaerobic plaque bacteria cause an adverse effect on peri-implant tissue health; bacteria proliferation in biofilms adhering to the implant surfaces can lead to peri-implantitis [16,17,18,19]. The overall increased frequency of peri-implantitis suggests that bacteria—mainly *Porphyromonas gingivalis, Fusobacterium* spp., and *Prevotella intermedia* [20,21]—play a role in causing damage to oral tissues [22,23]. Patients with peri-implantitis show different levels of bone loss around the implant; the most severe bone loss eventually results in implant loss. Bacterial infection has a major role in dental implant failure. Therefore, to date, effective inhibition and minimization of bacterial growth within dental biofilm on implant surfaces has been a priority [24].

To understand how to reduce the incidence of peri-implantitis, many studies have focused on infection reduction methods, including prevention of pathogen accumulation during plaque and calculus formation [25,26,27]. Few reports on implant surface treatments have shown that metal curettes and conventional ultrasonic scalers have caused less damage to the surface [28,29,30,31], but elimination of plaque and calculus has remained incomplete when dental implants have rough surfaces [28,32]. The use of lasers has been more effective than conventional approaches because of better removal of biofilm and the bacteria within it, in combination with other advantages [26]. Erbium, chromium-doped yttrium, scandium, gallium, and garnet (Er,Cr:YSGG) lasers are used on hard tissue during clinical dental surgery [33]. In general practice, it is used on soft tissue lesions, tooth structure, and bone because Er,Cr:YSGG is well-absorbed by collagen, hydroxyapatite, and water components. Because of these properties, the Er,Cr:YSGG laser has been applied in patients with peri-implant mucositis and peri-implantitis and has produced good soft tissue benefits and less postoperative pain and swelling than have conventional approaches [34,35]. During the initial bacterial infection process, biofilm on the implant surface helps bacteria form colonies, which can be disrupted and eliminated by lasers [36]. For those reasons, we aimed to evaluate the effects of Er,Cr:YSGG laser on Ti disc surfaces and on bacteria adhesion and colony formation, along with fibroblast viability and osteoblast differentiation.

## 2. Materials and Methods

To examine Er,Cr:YSGG laser effects on Ti disc surfaces, *P*. *gingivalis* adhesion, and fibroblasts, we induced colony formation on grade II 10 × 1 mm Ti alloy discs with smooth surfaces (BioTech One, Inc., Taipei, Taiwan). The discs were divided into two groups: control discs and Er,Cr:YSGG laser–treated discs.

### 2.1. Titanium Disc Preparation

Before the tests, Ti discs were ultrasonically cleaned in a detergent solution for 15 min, followed by rinsing with pure distilled water (Milli-Q; Millipore, Bedford, MA, USA) for 15 min. Then all discs were ultrasonically cleaned with acetone (product no. 650501; Sigma Aldrich Corp., St. Louis, MO, USA) for 15 min and were subsequently rinsed twice for 15 min each with pure distilled water. After the cleaning, Ti discs were sterilized by autoclaving at 121 °C for 20 min and dried at 40 °C in a conventional oven. The discs were then either autoclaved alone (control group) or autoclaved and then treated with Er,Cr:YSGG laser (test group).

### 2.2. Er,Cr:YSGG Laser Treatment

Samples were fixed by silicones (Aquasil Soft Putty; Dentsply Sirona, York, PA, USA) on a plate and exposed to the laser (LiteTouch Dental Laser; Syneron Dental Lasers, Yokneam Illit, Israel). In this study, we used a laser length of 2780 nm at 1.50 W, 20 Hz, with 11% distilled water and 7% air. The laser tip moved in a zigzag pattern at a distance of 2 mm from the disc, during 30 s of radiation treatment on the Ti disc surfaces.

### 2.3. Scanning Electron Microscopy

To investigate Ti disc structure, the discs were sputter coated with gold nanoparticles. Specimens were photographed and analyzed in a fully computerized, high-resolution scanning electron microscope (SEM) with 20-kV power (SU3500; Hitachi Ltd., Kyoto, Japan). Five random digital images were taken of each control disc and each laser–treated disc at 500 times magnification.

### 2.4. Energy-Dispersive Spectrometry

Ti disc surfaces were analyzed by energy-dispersive spectrometry (EX-250; HORIBA, Kyoto, Japan) with an electron beam covering a 70-µm spectrum and an accelerating voltage of 15 kV to derive the surface elemental composition of the surfaces of control and Er,Cr:YSGG laser–treated discs.

### 2.5. X-ray Photoelectron Spectroscopy

We used X-ray photoelectron spectroscopy (ESCA system; VG Scientific, West Sussex, UK) with a 1486.6-eV monochromatic X-ray source for elemental and chemical analyses of treated surfaces. Spectra were collected at an electron takeoff angle normal to the disc surfaces.

### 2.6. Examination of Surface Wettability by Contact Angle

The contact angle was measured with the sessile drop method and a plane goniometer (Digidrop Goniometer; GBX, Romans sur Isère, France). A 4-μL droplet of distilled water (filtered, 20 °C; Milli-Q, Millipore) was applied to the surface of each disc, and wettability was measured [37].

### 2.7. Examination of Surface Roughness

To characterize the topography of the Ti discs before and after Er,Cr:YSGG laser treatment, the profile roughness parameter, a measure of mean surface roughness on control and Er,Cr:YSGG laser–treated discs, was evaluated with a profilometer (TR200; An-Bomb Instrument Co., Ltd., Tainan, Taiwan).

### 2.8. Fibroblast Cell Culture

Green fluorescent protein labeled NIH/3T3 fibroblasts (CRL-1658; LGC Standards, Teddington, UK) were seeded at a density of 1.5 × 10^5^ cells/cm^2^ in plastic tissue culture dishes (Falcon; Becton Dickinson, NJ, USA), containing Dulbecco’s modified Eagle’s medium (Sigma Aldrich), supplemented with 10% fetal bovine serum (Gibco BRL, Rockville, MD, USA) and antibiotics (penicillin/streptomycin; Gibco BRL) in a fully humidified atmosphere consisting of 95% room air and 5% CO_2_ at 37 °C [38,39].

### 2.9. Cell Cytotoxicity

For fibroblast cytotoxicity assessment, the cells (1.5 × 10^4^ cells/disc) were seeded onto control and Er,Cr:YSGG laser treated disc surfaces in 12-well plates (Falcon) and incubated for 1, 3, and 5 days. After washing with phosphate buffered solution, 10 µL of water-soluble tetrazolium 1 (WST-1) reagent (WST-1 Kit; Roche Applied Science, Mannheim, Germany) was added to each well, and the wells were incubated for 4 h at 37 °C in an atmosphere of 95% room air and 5% CO_2_. Next, samples were placed in 96-well plates and shaken for 1 min to mix the contents. Subsequently, the absorbance of the samples was measured with a microplate reader at the optical density of 450 nm with a reference wavelength of 650 nm [40].

### 2.10. ALP Activity

Alkaline phosphatase (ALP) activity was measured after cells (1.5 × 10^4^ cells/disc) were seeded onto control and Er,Cr:YSGG laser–treated disc surfaces in 12-well plates (Falcon) and incubated for 1, 3, and 5 days. We used highly sensitive, simple, direct and high-throughput screening ready colorimetric assay designed to measure ALP activity (Alkaline Phosphatase Activity Colorimetric Assay Kit, catalog no. 412-500; BioVision Inc., Milpitas, CA, USA). The absorbance of the samples was measured with a microplate reader at the optical density of 405 nm with a reference wavelength of 650 nm.

### 2.11. Porphyromonas Gingivalis Adhesion

Standard reference strain *P*. *gingivalis* ATCC 33277 was selected for this study (Microbiologics Ltd., St. Cloud, MN, USA). Bacteria was cultured in ATCC Medium: 260 Tryptic Soy Agar/Broth for 72 h under anaerobic conditions (10% H_2_, 10% CO_2_, and 80% N_2_ at 37 °C). Subsequently, this medium was used to dilute the bacterial concentration to 10^6^ colony-forming units (CFUs)/mL of broth. Control and Er,Cr:YSGG laser treated discs previously placed individually in 12-well plates were completely immersed in 3 mL of the medium and maintained in a shaker incubator at 60 °C and 75 rpm from 1 to 5 days. For each analyzed Ti disc, we inspected the maximum absorbance by scanning with a spectrophotometer (GENESY 10S Series Spectrophotometer; Thermo Fisher Scientific, Inc., Waltham, MA, USA) at an optical density of 660 nm.

### 2.12. Statistical Analysis

All experimental values are presented as means ± standard deviation of at least three independent recordings. Data were analyzed with SPSS software (IBM Corp., Armonk, NY, USA) and Excel (Microsoft, Inc., Redmond, WA, USA).

## 3. Results

### 3.1. Er,Cr:YSGG Laser Treated Ti Discs Surface Characterization by SEM Image

**Figure 1:**
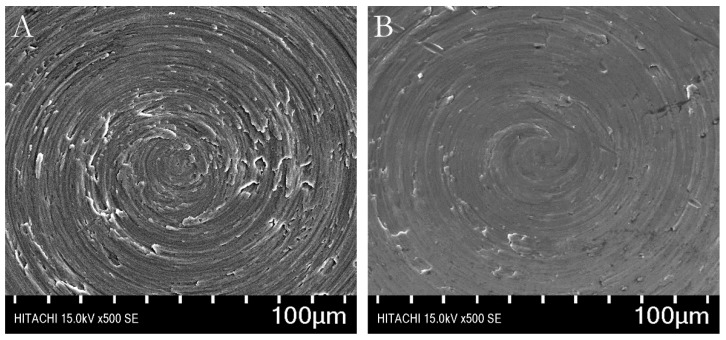
Scanning electron microscopic images of titanium disc surfaces. (**A**) A typical control (autoclaved) disc. (**B**) A typical test disc treated with erbium, chromium-doped yttrium, scandium, gallium, and garnet (Er,Cr:YSGG) laser. (magnification 500×).

All disc surfaces had a predominantly circular pattern. The Er,Cr:YSGG laser treated Ti discs had slightly less roughness than did the autoclaved control discs (Figure 1).

### 3.2. Energy-Dispersive Spectrometry

**Figure 2:**
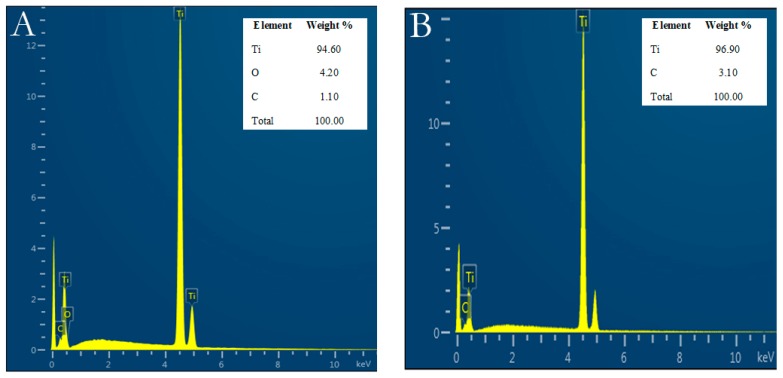
Titanium (Ti) weight percentage on control discs and on test discs before and after erbium, chromium-doped yttrium, scandium, gallium, and garnet (Er,Cr:YSGG) laser treatment. (**A**) Control discs. (**B**) Er,Cr:YSGG laser–treated discs.

Ti element was found in both control and Er,Cr:YSGG laser–treated Ti discs in almost the same weight percentages, which were not statistically significant different. Control discs had 94.60 Ti wt%, 4.20 oxygen wt%, and 1.10 carbon wt%; while Er,Cr:YSGG laser–treated Ti discs had 96.90 Ti wt% and 3.10 carbon wt% (Figure 2).

### 3.3. X-ray Photoelectron Spectroscopy Examination of Surface Elements

**Figure 3:**
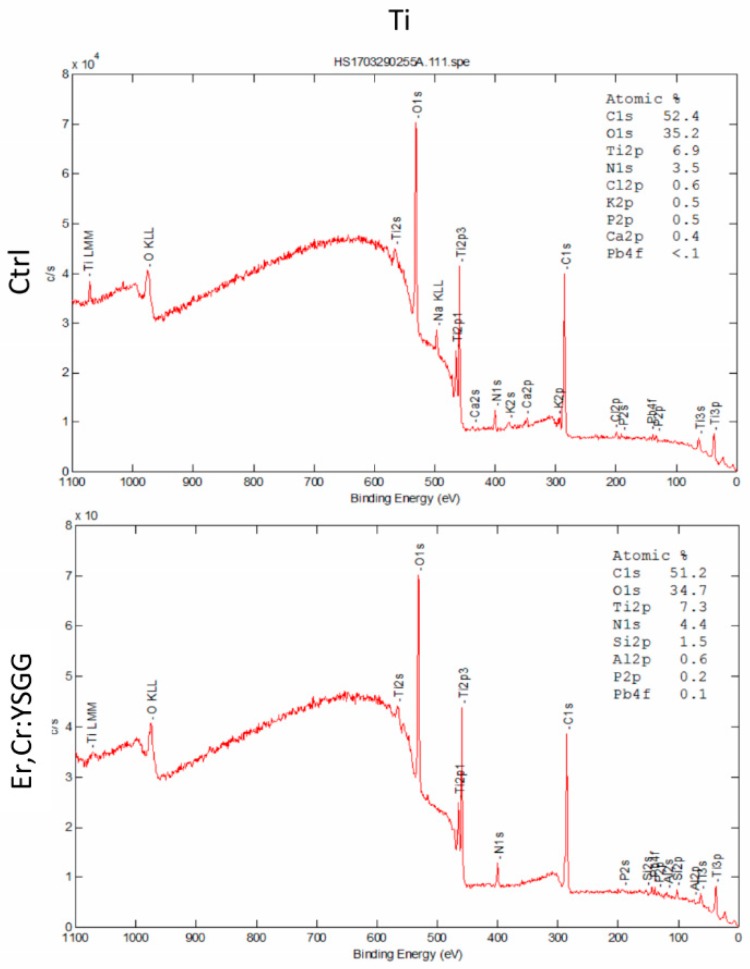
X-ray photoelectron spectra spectrum, showing the atomic ratio of the control (Ctrl) autoclaved discs and the Er,Cr:YSGG: erbium, chromium-doped yttrium, scandium, gallium, and garnet laser–treated (test) discs.

The chemical constituents of the surface of the Ti discs were analyzed by X-ray photoelectron spectroscopy, which revealed that the carbon (C1s) values were similar for control and Er,Cr:YSGG laser–treated Ti discs (52.4% and 51.2%, respectively); the oxygen (O1s) values were 35.2% for control discs and 34.7% for Er,Cr:YSGG laser–treated Ti discs; and the titanium (Ti2p) values were 6.9% for control discs and 7.3% for Er,Cr:YSGG laser–treated Ti discs in Figure 3. Thus, the control discs and test discs had approximately the same percentages of elements in their surface structures.

### 3.4. Wettability of Titanium Disc Surface

**Figure 4:**
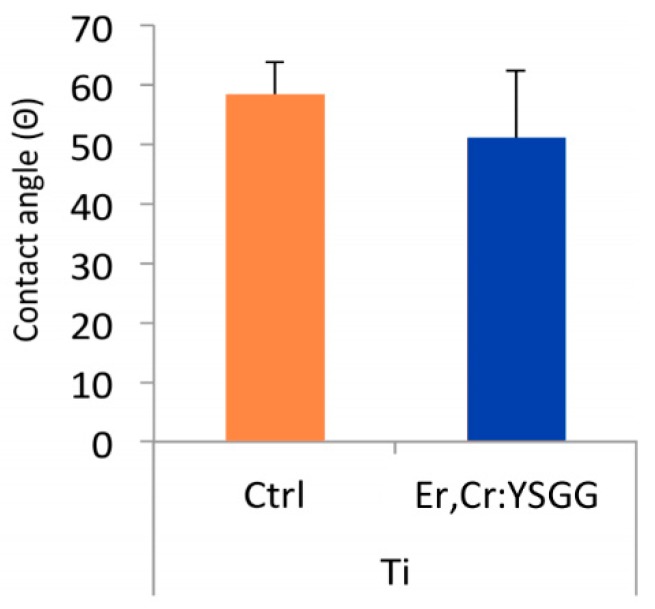
Measurement of contact angle on treated implant surface. Analysis indicated surface wettability of the erbium, chromium-doped yttrium, scandium, gallium, and garnet (Er,Cr:YSGG) laser–treated titanium (Ti) discs was better than that of the control (Ctrl) samples.

Surface wettability was analyzed via a contact angle measurement (Figure 4). The average contact angles in Er,Cr:YSGG laser–treated Ti discs (50.93 ± 19.74 degrees) were smaller than those in control Ti discs (58.23 ± 9.56 degrees), which indicates that Er,Cr:YSGG laser–treated surfaces had superior hydrophilicity with high surface wettability.

### 3.5. Roughness of Titanium Disc Surface

**Figure 5:**
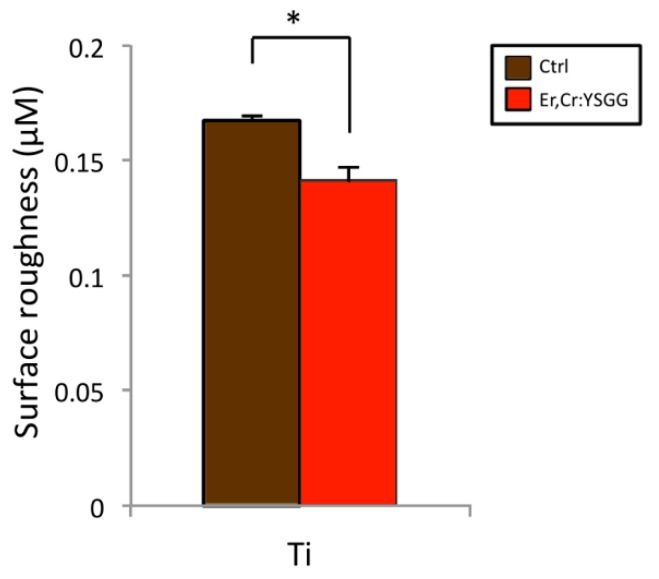
Effects on the surface roughness of control (Ctrl) titanium (Ti) discs and of test Ti discs treated with erbium, chromium-doped yttrium, scandium, gallium, and garnet (Er,Cr:YSGG) laser. Test discs showed slightly reduced roughness after Er,Cr:YSGG laser treatment in comparison with control discs. * *p* < 0.05.

Quantitative analysis of surface roughness indicated that changes in the profile roughness parameter were significantly different in the two disc groups (Figure 5). Values of control Ti discs were 0.167 ± 0.004 μm, and those of Er,Cr:YSGG laser–treated Ti discs were 0.142 ± 0.005 μm (*p* < 0.05).

Er,Cr:YSGG laser–treated disc surfaces: increased fibroblast viability and osteoblastic differentiation.

**Figure 6:**
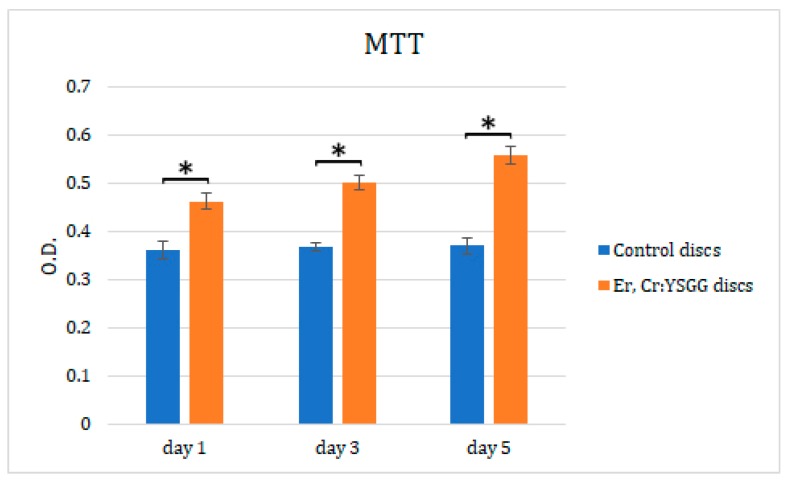
Effects of erbium, chromium-doped yttrium, scandium, gallium, and garnet (Er,Cr:YSGG) laser–treated Ti discs on NIH/3t3 fibroblast cell viability, according to MTT assay. Cells were incubated from day 1 to 5. Statistically significant differences were found on all days. * *p* < 0.05.

Cell cytotoxicity assay results (Figure 6) showed a clear trend of greater fibroblast proliferation on Er,Cr:YSGG laser–treated Ti discs (optical density values of 0.46 ± 0.015 on day 1, 0.5 ± 0.016 on day 3, and 0.56 ± 0.017 on day 5) than did control discs, on which fibroblasts had lower viability (optical density values of 0.46 ± 0.015 on day 1, 0.5 ± 0.016 on day 3, and 0.56 ± 0.017 on day *5 days; all Ps < 0.05).* In addition, the viability of fibroblasts on Er,Cr:YSGG laser–treated Ti discs increased from day 1 to day 5, whereas that of fibroblasts cultivated on control discs was similar on all days (Figure 6).

**Figure 7:**
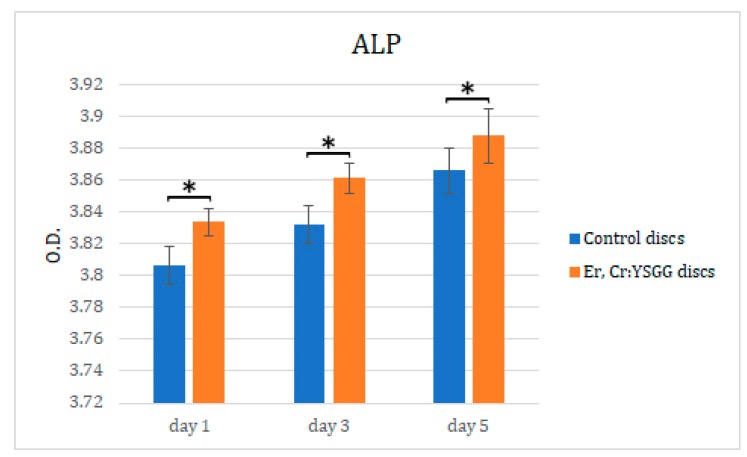
Effects of erbium, chromium-doped yttrium, scandium, gallium, and garnet (Er,Cr:YSGG) laser–treated Ti discs on NIH3t3 fibroblast alkaline phosphatase (ALP) activity. Cells were incubated from day 1 to 5. O.D., optical density. Statistically significant differences were found in all days. * *p* < 0.05.

Er,Cr:YSGG laser treatment on disc surfaces influenced fibroblasts osteoblastic differentiation, which we assessed by measuring ALP activity. As shown in Figure 7, there was a higher increase in ALP expression in cells cultured on Er,Cr:YSGG laser–treated discs on days 1, 3, and 5 (optical densities of 3.83 ± 0.008, 3.86 ± 0.009, and 3.89 ± 0.017, respectively). The fibroblasts cultured on control discs showed optical densities of 3.81 ± 0.012 on day 1, 3.83 ± 0.012 on day 3, and 3.87 ± 0.014 on day 5; they also had less osteoblastic differentiation than did fibroblasts on Er,Cr:YSGG laser treated discs (*p* < 0.05). These results strongly suggest that Ti disc surfaces directly stimulated the osteoblastic differentiation of NIH/3T3 fibroblasts cells in Vitro.

### 3.6. Porphyromonas Gingivalis Adhesion and Colony Formation On Titanium Discs after Er,Cr:YSGG Laser Treatment

**Figure 8:**
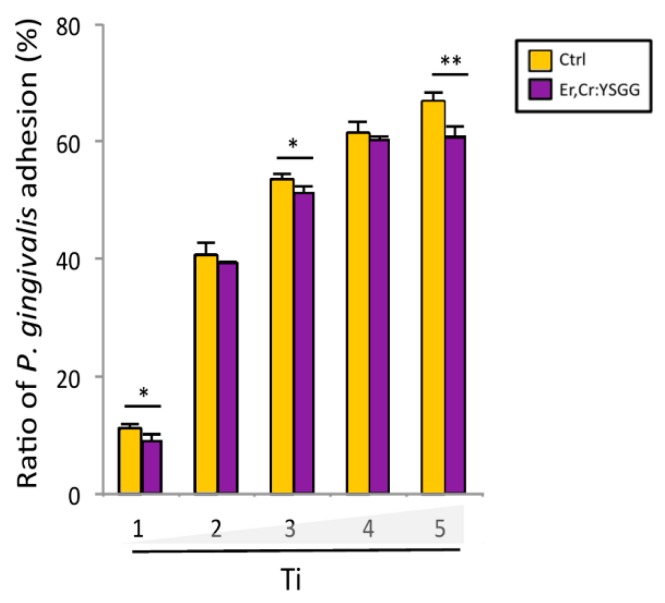
Adhesion of *Porphyromonas gingivalis* on titanium (Ti) discs. At each time point, the discs treated with erbium, chromium-doped yttrium, scandium, gallium, and garnet (Er,Cr:YSGG) laser demonstrated lower bacteria adhesions than did control (Ctrl) discs. * *p* < 0.05, ** *p* < 0.01.

**Figure 9:**
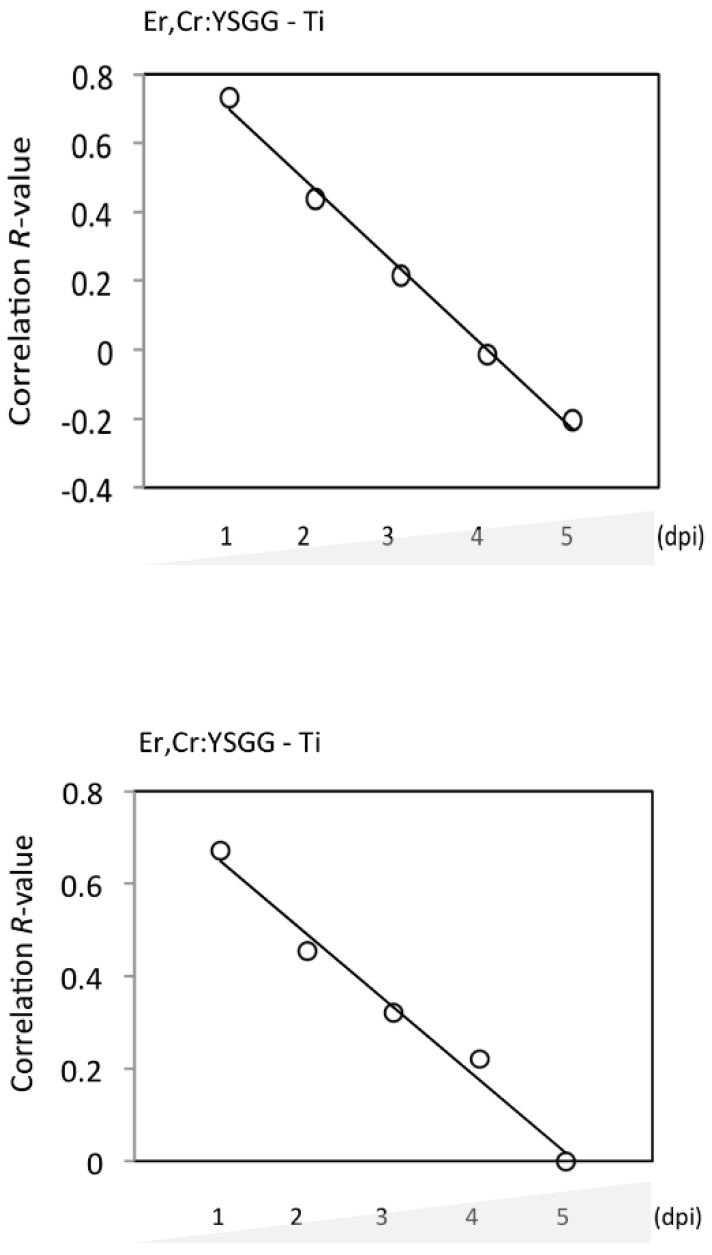
*R* correlation between erbium, chromium-doped yttrium, scandium, gallium, and garnet (Er,Cr:YSGG) laser treatment effectively reducing bacteria adhesion (**A**) and colony formation (**B**) on titanium (Ti) discs.

*P*. *gingivalis* adhesion measured at an optical density of 660 nm indicated that Er,Cr:YSGG laser–treated discs had bacteria adhesion rates of 9.11% ± 2.32% on day 1, 39.59% ± 0.33% on day 2, 51.35% ± 2.34% on day 3, 60.46% ± 1.69% on day 4, and 60.79% ± 3.98% on day 5. Control discs had bacteria adhesion rates of 11.16% ± 1.69% on day 1, 40.75% ± 4.36% on day 2, 61.62% ± 3.33% on day 3, 67.25% ± 2.98% on day 4, and 67.25% ± 2.98% on day 5. These results show that *P*. *gingivalis* adhesion was slightly lower on Er,Cr:YSGG laser–treated discs than on control discs; the differences were statistically significant on days 1, 3, and 5 (Figure 8). During the 5-day test, the correlation between surface roughness of Er,Cr:YSGG laser–treated discs and bacteria adhesion decreased gradually in a time-dependent manner (Figure 9A). Thus Er,Cr:YSGG laser treatment was associated with lower *P*. *gingivalis* adhesion.

**Figure 10:**
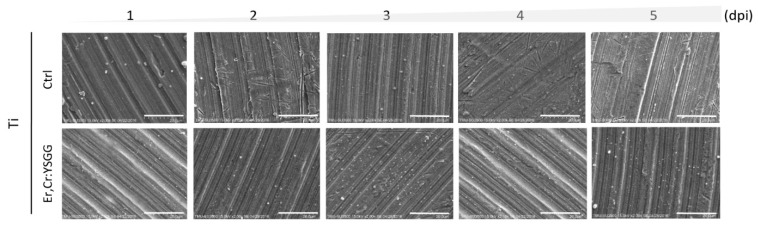
Qualitative morphological scanning electron microscopic (SEM) micrographs of *Porphyromonas gingivalis* on control (Ctrl) titanium (Ti) discs and on Ti discs after erbium, chromium-doped yttrium, scandium, gallium, and garnet (Er,Cr:YSGG) laser treatment. These images illustrate the growth of bacteria and show relatively fewer bacteria on the Er,Cr:YSGG laser–treated discs. Magnification, 2000 times; scale bar = 20 μm.

**Figure 11:**
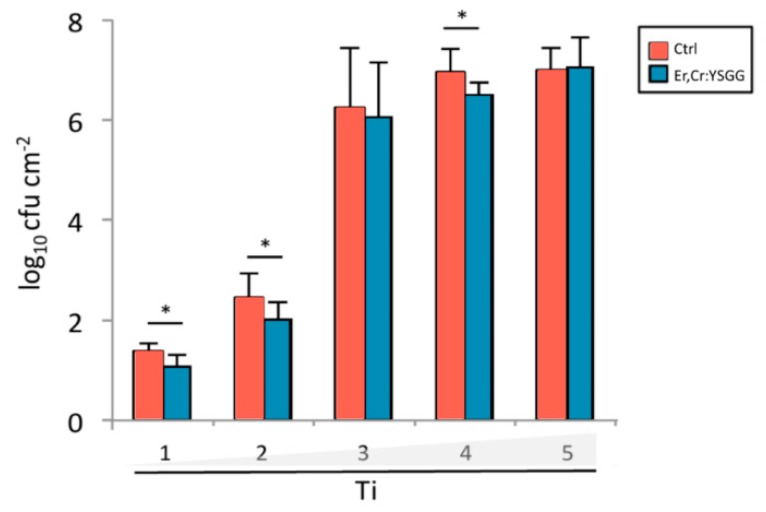
Graph of *Porphyromonas gingivalis* colony formation on control (Ctrl) titanium (Ti) discs and on Ti discs after erbium, chromium-doped yttrium, scandium, gallium, and garnet (Er,Cr:YSGG) laser treatment. * *p* < 0.05.

Scanning electron microscopic (SEM) analysis on Ti discs were done to determine the extent of bacteria colony formation from days 1 to 5. Figure 10 depicts qualitative views, in which Er,Cr:YSGG laser–treated Ti discs demonstrated moderately fewer bacterial colonies than did the control discs. During 5 days of observation, the quantified data showed a remarkable increase in *P*. *gingivalis* accumulation after 2 dpi (Figure 11). Control discs had 91.37 ± 0.30 CFUs on day 1, 2.44 ± 0.90 CFUs on day 2, 6.27 ± 2.40 CFUs on day 3, 6.99 ± 0.98 CFUs on day 4, and 7.02 ± 0.90 CFUs on day 5. Er,Cr:YSGG laser–treated Ti discs had 1.05 ± 0.47 CFUs on day 1, 2.03 ± 0.68 CFUs on day 2, 6.08 ± 2.18 CFUs on day 3, 6.52 ± 0.51 CFUs on day 4, and 7.08 ± 1.24 CFUs on day 5 (Figure 11). These findings demonstrate that slightly fewer *P*. *gingivalis* colonies formed on Er,Cr:YSGG laser treated discs than on control discs; the differences were statistically significant on days 1, 2, and 4. During the 5-day period, the correlation between the surface roughness of Er,Cr:YSGG laser treated discs and bacterial colony formation decreased gradually in a time-dependent manner (Figure 9B).

## 4. Discussion

This study showed that Er,Cr:YSGG laser reduced bacterial adhesion and increased fibroblast adhesion on dental implant material, with subsequent smoother surfaces that interrupted the initial phase of adhesion. Both peri-implant mucositis and peri-implantitis have been related to biofilm accumulation, and so the removal of this biofilm and the bacteria within it is important for the longevity of dental implants. Implant surfaces have gone through an evolutionary change from machined titanium to rough surfaces. Rough implant surfaces enhances implant stability in bone, but they may provide an environment conducive to increased plaque and bacterial colonization and thereby result in peri-implantitis [24].

Many techniques have been used to treat peri-implantitis, including conventional methods such as mechanical debridement, chemical surface treatment, and laser therapy [41]. The Er,Cr:YSGG laser used in this study was set at a wavelength of 2780 nm with 1.50 W, 20 Hz, 11% distilled water, and 7% air, and the laser tip was 2 mm away from the disc and applied for 30 s on the disc surfaces. In these settings, the laser treatment ablated tissue by a hydrokinetic process that prevents temperature increase. As in Azzeh et al.’s [24] report, the Er,Cr:YSGG laser used in this study was highly efficient and effective in removing contaminants from the implant material with minimal changes to the titanium surface roughness and the lack of an organic smear layer In Vitro. Our findings have shown that Ti implant materials can be treated with Er,Cr:YSGG to smooth surfaces and interrupt *P. gingivalis* adhesion. Thus Er,Cr:YSGG treatment can be considered a useful method for dental implant decontamination.

According to our results, Er,Cr:YSGG slightly modified pure titanium disc surfaces by making them less rough (Figure 1) while maintaining the Ti surface weight percentage (Figure 2). Over a period of 5 days, *P. gingivalis* attachment was reduced. The X-ray photoelectron spectroscopy (Figure 3) indicated that Er,Cr:YSGG laser–treated Ti discs had the same concentrations of elements as the control discs (Figure 4), despite having less roughness of the surface (Figure 5). As SEM analysis showed, we found no signs of any laser-induced thermal side effects such as melted areas because of temperature’s surpassing the metallic melting and boiling thresholds (Figure 10). As previously reported, wettability is directly proportional to surface roughness; therefore, Er,Cr:YSGG laser–treated Ti discs were slightly less wettable than control discs, but the difference was not significantly different (Figure 4).

Er,Cr:YSGG laser–treated Ti discs had less bacteria adhesion 5 days after treatment than did the control discs (*p* < 0.01; Figure 8). Similar results were found in the quantitative SEM analysis of *P. gingivalis,* in which the bacterial adhesion was significantly different at 4 days (Figure 10 and Figure 11). Although bacterial culture revealed less bacterial adhesion on Er,Cr:YSGG laser–treated Ti discs, whereas SEM analysis showed little difference at 4 days, both test results indicated a tendency for less bacteria on Er,Cr:YSGG laser treated Ti discs than on control discs on the first days. This tendency was found in an *R* correlation between surface roughness and adherence of less bacteria to the Er,Cr:YSGG laser treated Ti discs in both the culture and SEM analysis (Figure 9). We believe the slight difference between the results of these two analyses is attributable to the fact that the area examined in the SEM analysis was smaller than that used in the culture test.

Laser irradiation at 100 mJ/pulse and 10 Hz for 1 min is suggested as a standard for detoxification of implant surfaces [42,43]. In our study, the use of 1.50 W, 20 Hz, 11% distilled water, and 7% air with a laser tip–to–disc distance of 2 mm and 30 s of irradiation effectively inhibited *P. gingivalis* adhesion and maintained the Ti surface. These results are important for the implant–mucosa interface, where numerous implants have a polished collar, similar in roughness and elements to the Ti discs used in this study, and where biofilm accumulates, which can lead to peri-implant mucositis and the potential for peri-implantitis. Our Er,Cr:YSGG laser settings could be used to treat peri-implant mucositis and peri-implantitis with minimal implant surface alteration.

Schwarz et al. [44], using the same Er,Cr:YSGG laser machine as in this study, with settings of 1.5 W and 20 Hz with an air/water ratio of 50/50, demonstrated high efficiency in removing plaque biofilm in an energy-dependent manner, but they did not reestablish the biocompatibility of contaminated titanium surfaces. We did measure cell viability and osteoblast differentiation (Figure 6 and Figure 7) and found a greater increase in fibroblasts on the Ti discs treated with Er,Cr:YSGG laser than in cells cultured on control discs (*p* < 0.05). Furthermore, fibroblasts also exhibited higher osteoblast differentiation when cultured on Ti discs treated with Er,Cr:YSGG laser (*p* < 0.05). These findings are in accordance with those of Gheith et al. [45], who, after cell viability testing and SEM analysis, concluded that Er,Cr:YSGG laser could safely improve the biocompatibility of dental implants made of titanium alloy (Ti-6Al-4V); no undesirable changes were observed with the use of 2 W. Other studies showed surface alterations such as melting and flattening with settings of 2 W, 20 Hz, 4 mm, and 45 s, and with settings of 3 W, 25 Hz, 2 mm, 45 s on Ti discs; these settings were higher than those used in this study [46]. These outcomes suggest that laser parameters, such as the ones used in this study, should be optimized to conserve the maximum surface characteristics possible during the irradiation of implant surfaces.

As Miranda et al. [47] pointed out, the wavelength of the Er,Cr:YSGG laser is highly specific to water and is sensitive to gingival fluid, saliva, and blood. This makes the process of clinical implant surface decontamination different from that in in-vitro situations. Other clinical studies have shown good clinical results that support our results. Soldatos et al. [48] described a case of dental implant stability 13 months after Er,Cr:YSGG laser treatment for peri-implantitis, showing increased radiographic bone density. Other studies have demonstrated that the Er,Cr:YSGG laser is more advantageous than mechanical therapy, such as that with titanium curettes or citric acid, in the treatment of peri-implantitis [49,50]. Therefore, it is important to determine the best laser settings, such as the power and the distance between the tip and titanium surface so as to achieve safer guidelines for clinicians in decontamination, promoting biofilm removal, reducing bacterial adhesion, and improving cell attachment, while the dental implant surface characteristics are preserved after laser irradiation [46].

Some of the limitations of this study involve not demonstrating the possible application of Er,Cr:YSGG for decontamination of implant surface colonized by *P. gingivalis.* In further studies, laser treatment should be applied after bacteria culture.

## 5. Conclusions

Er,Cr:YSGG laser treatment made Ti disc surfaces slightly smoother. According to the results, reduction of *P. gingivalis* adhesion and increasing of fibroblast viability and osteoblast differentiation were observed with Er,Cr:YSGG laser treatment. Er,Cr:YSGG laser treatment altered the Ti surface properties and improved the biological responses to the treated surfaces. Therefore, Er,Cr:YSGG laser treatment can be considered for laser-assisted therapy settings and in guidelines for the treatment of peri-implantitis.
